# Supplementary material for: Impact of the COVID‐19 Economic Downturn on Tropospheric Ozone Trends: An Uncertainty Weighted Data Synthesis for Quantifying Regional Anomalies Above Western North America and Europe
Source: AGU Adv. 2022 Mar 4;3(2):e2021AV000542. doi: 10.1029/2021AV000542 (PMC9111294; doi:10.1029/2021AV000542)
Supplement: Supplementary file 1 — Supporting Information S1 [file AGA2-3-0-s002.pdf]

AGU Advances

Supporting Information for

Impact of the COVID-19 economic downturn on tropospheric ozone trends: an uncertainty weighted data synthesis for quantifying regional anomalies above western North America and Europe

Kai-Lan Chang[1,2], Owen R. Cooper[1,2], Audrey Gaudel[1,2], Marc Allaart[3,] Gerard Ancellet[4], Hannah Clark[5], Sophie Godin-Beekmann[4], Thierry Leblanc[6], Roeland Van Malderen[7], Philippe Nédélec[8], Irina Petropavlovskikh[1,9], Wolfgang Steinbrecht[10], René Stübi[11], David W. Tarasick[12], Carlos Torres[13]

[1] Cooperative Institute for Research in Environmental Sciences, University of Colorado Boulder, CO, USA

[2] NOAA Chemical Sciences Laboratory, Boulder, CO, USA

[3] Royal Netherlands Meteorological Institute, De Bilt, The Netherlands

[4] LATMOS, Sorbonne Université-UVSQ-CNRS/INSU, Paris, France

[5] IAGOS-AISBL, 98 Rue du Trône, Brussels, Belgium

[6] Jet Propulsion Laboratory, California Institute of Technology, Wrightwood, CA, USA

[7] Royal Meteorological Institute of Belgium, Uccle, Belgium

[8] Laboratoire d'Aérologie, CNRS and Université de Toulouse III, Toulouse, France

[9] NOAA Global Monitoring Laboratory, Boulder, CO, USA

[10] Deutscher Wetterdienst, Hohenpeiβenberg, Germany

[11] Federal Office of Meteorology and Climatology, MeteoSwiss, Payerne, Switzerland

[12] Environment and Climate Change Canada, Toronto, ONT, Canada

[13] Izaña Atmospheric Research Center, AEMET, Tenerife, Spain

List of Tables

S-1 Ozone trends in different pressure layers. Trend values and 2-sigma uncertainty [in units of ppbv/decade] are based on monthly means and linear regression models. The relative change [%] of trends is based on the absolute value of the trend value through the end of 2019. Trends with a magnitude greater than 2-sigma are shown in bold font, and trends with a magnitude greater than 3-sigma are shown in bold and italic. Reference years for each station are listed in Table 2. . . . 3
S-2 Same as Table S1. . . . 4
S-3 Same as Table S1. . . . 5

List of Figures

S-1 A map view of ozonesonde or lidar stations (red circles), and the major airports visited by IAGOS aircraft in Europe and western North America (blue boxes). . . . 6
S-2 Comparisons of ozone time series at Kelowna (2003-2017) and Port Hardy (2018-2020) based on the 95th, 50th and 5th percentiles at different pressure layers. . . . 7
S-3 Residuals diagnostics of the tropospheric ozone data fusion above Western Europe. . . . 8
S-4 Ozone mean distributions above Western Europe based on the normalized deviations. . . . 9
S-5 Same as Figure S4, but ozone mean distributions are transformed back to the units of ppbv. . . . 10
S-6 Relative variability (RV) from each ozonesonde record and IAGOS data above Western Europe. The estimations are based on the standard error associated with each monthly aggregated mean divided by the overall mean at the corresponding pressure surface. . . . 11
S-7 A demonstration of the influence of stratosphere-troposphere exchange on tropospheric ozone trends above Europe over 1994-2018. The first row shows the vertical distribution and trends, when the stratospheric air masses are included. The second row is the same as the first row, but the stratospheric air masses are removed. . . . 12
S-8 A demonstration of the influence of diurnal cycle on tropospheric ozone trends above Europe, by comparing the trends in the European IGAOS data set with and without the nighttime observations. . . . 13
S-9 Ozone mean distributions above Uccle over 1969-2020 based on normalized deviation [ND] and the units of ppbv. . . . 14
S-10 Comparison of monthly anomalies from ozonesondes and surface measurements limited within the cold (Oct-Apr) or warm (May-Sep) season at different pressure surfaces in Western Europe. The overall trends are based on the simple average of ozonesonde and IAGOS data (not including the surface measurements). 15
S-11 Comparisons of monthly anomalies from ozonesonde and surface measurements limited within the cold (Oct-Apr) or warm (May-Sep) season at different pressure surfaces (1970-2020). The smooth curves represent the Loess fits and their 2-sigma intervals. . . . 16
S-12 Ozone mean trends above Western Europe [in units of ppbv/decade] derived from the fused product over 2004-2019 (left panel) and 2008-2019 (right panel). . . . 17
S-13 Ozone mean distributions above western North America based on the normalized deviations. . . . 18
S-14 Same as Figure S13, but ozone mean distributions are transformed back to the units of ppbv. . . . 19
S-15 Relative variability (RV) from each ozonesonde record and IAGOS data above western North America. The estimations are based on the standard error associated with each monthly aggregated mean divided by the overall mean at the corresponding pressure surface. . . . 20
S-16 Ozone mean trends above western N. America [in units of ppbv/decade] derived from the fused product over different periods. . . . 21
S-17 Detailed inspection of 2020 ozone anomalies [in units of ppbv] above western Europe and western North America, limited to the period of 2015-2020. . . . 22
S-18 Ozone mean distributions based on the normalized deviations. . . . 23
S-19 Same as Figure S18, but ozone mean distributions are transformed back to the units of ppbv. . . . 24

S-1 Statistical background for the penalized least square criterion

The penalized least square criterion shown in Equation (4) of the main text can be seen as a combination of a (weighted) residual sum of squares (aiming to quantify the goodness of fit) and a roughness penalty $D^2\mathbf{F}$ (aiming to avoid over-fitting). The roughness penalty essentially controls the smoothness of a fitted surface (in our case, the ozone vertical distribution) and can be defined as (Wood, 2006):

$$\begin{aligned} D^2\mathbf{F} =& \lambda \iint \left[\frac{\partial^2 f(h,t)}{\partial t^2}\right]^2 + \left[\frac{\partial^2 f(h,t)}{\partial h^2}\right]^2 + 2\left[\frac{\partial^2 f(h,t)}{\partial h \partial t}\right]^2 \mathrm{d}h\mathrm{d}t \\ &+ \lambda_s \iint \left[\frac{\partial^2 g_s(h,t)}{\partial t^2}\right]^2 + \left[\frac{\partial^2 g_s(h,t)}{\partial h^2}\right]^2 + 2\left[\frac{\partial^2 g_s(h,t)}{\partial h \partial t}\right]^2 \mathrm{d}h\mathrm{d}t, \end{aligned} \tag{1}$$

where $\{\lambda, \lambda_s\}$ are the tuning parameters for the consensus process and structured discrepancy (if any) from station s, respectively.

S-2 Additional analysis

The rich dataset compiled for Europe has relevance to other recent studies as follows:

1. Cooper et al. (2020) recently compared ozone trends at the three high elevation, long-term ozone monitoring sites in the Alps (Jungfraujoch, Zugspitze and Sonnblick) to the IAGOS trends to demonstrate that the Alpine sites are impacted by the European boundary layer and are not fully representative of the lower free troposphere, especially in summer. Figure S10 expands this comparison by including the ozonesonde sites of Hohenpeissenberg, Haute-Provence, Payerne and Uccle.

2. Gaudel et al. (2018) compared trends of tropospheric column ozone among five satellite products based on either the OMI (2004-2016) or IASI (2008-2016) satellite instruments. Hemispheric scale trends diverged among some of the products after 2008. To allow for detailed evaluation of these products above Europe, where ozone profiles are most abundant, we report ozone trends from the surface to the upper troposphere for the periods 2004-2019 and 2008-2019 (Figure S12) above Europe.

   Whereas certain similarity of trends can be found above Europe from 2004 or 2008, the trends above western North America are more diverse. Figure S16 shows the western North America trends beginning in different years, demonstrating that the trends above this region are highly sensitive to the starting year, presumably due to limited sample sizes (including the IAGOS data set) and monitoring stations that are spread across a larger region. These limitations result in a trend assessment that is less robust above western North America than above western Europe.

3. Oltmans et al. (2013) raised this issue of the discrepancy between the HPB ozonesondes and the Zugspitze mountaintop (2800 m elevation) observations in the 1980s. Figure S11 further shows the comparison of the ozone cold and warm season time series (1970-2020) from 1) HPB ozonesonde data at 900 hPa and nearby Hohenpeissenberg daytime surface measurements (985 m elevation), 2) HPB ozonesonde data at 700 hPa and Zugspitze nighttime surface measurements, and 3) Payerne ozonesonde data at 650 hPa and Jungfraujoch nighttime surface measurements (3580 m elevation). Monthly surface ozone values at Zugspitze, Hohenpeissenberg and Jungfraujoch are from (Cooper et al., 2020).

Table S-1: Ozone trends in different pressure layers. Trend values and 2-sigma uncertainty [in units of ppbv/decade] are based on monthly means and linear regression models. The relative change [%] of trends is based on the absolute value of the trend value through the end of 2019. Trends with a magnitude greater than 2-sigma are shown in bold font, and trends with a magnitude greater than 3-sigma are shown in bold and italic. Reference years for each station are listed in Table 2.

		Through 2019		Through 2020		
Site	Pressure [hPa]	Trend [±2-σ]	p-value	Trend [±2-σ]	p-value	Change [%]
Edmonton	950-250	***-7.76 [±2.95]***	<0.01	***-7.35 [±2.77]***	<0.01	5
	700-300	***-3.87 [±2.29]***	<0.01	***-3.79 [±2.13]***	<0.01	2
	400-300	***-6.42 [±3.92]***	<0.01	***-5.97 [±3.65]***	<0.01	7
	650	-0.63 [±1.48]	0.39	-0.73 [±1.38]	0.29	-14
	950-800	-0.29 [±1.03]	0.58	-0.22 [±0.97]	0.64	21
Kelowna & Port Hardy	950-250	0.58 [±2.42]	0.64	-0.07 [±2.15]	0.94	-113
	700-300	1.54 [±2.31]	0.18	1.21 [±2.07]	0.25	-22
	400-300	0.08 [±5.24]	0.98	-0.83 [±4.63]	0.72	-1151
	650	**1.97 [±1.75]**	0.03	1.57 [±1.70]	0.07	-20
	950-800	-0.13 [±2.83]	0.93	-0.56 [±2.76]	0.68	-327
Trinidad Head	950-250	-1.43 [±1.69]	0.09	**-1.97 [±1.61]**	0.01	-37
	700-300	-1.38 [±1.91]	0.15	**-1.89 [±1.80]**	0.04	-37
	400-300	-2.68 [±5.18]	0.30	-3.47 [±4.81]	0.15	-30
	650	-0.78 [±1.11]	0.16	**-1.12 [±1.06]**	0.04	-43
	950-800	-0.75 [±0.88]	0.09	**-1.15 [±0.88]**	0.01	-52
Boulder	950-250	**-1.26 [±0.90]**	0.01	***-1.27 [±0.85]***	<0.01	-1
	700-300	***-1.38 [±0.79]***	<0.01	***-1.37 [±0.75]***	<0.01	1
	400-300	**-2.14 [±1.67]**	0.01	**-1.93 [±1.57]**	0.01	10
	650	***-1.24 [±0.74]***	<0.01	***-1.34 [±0.70]***	<0.01	-8
	950-800	0.54 [±1.25]	0.39	0.33 [±1.18]	0.57	-38
Table Mountain	600-250	0.91 [±2.37]	0.45	0.39 [±2.15]	0.72	-57
	600-300	1.07 [±2.21]	0.33	0.58 [±2.00]	0.56	-45
	400-300	**2.89 [±2.78]**	0.04	2.14 [±2.54]	0.09	-26
	650	-	-	-	-	-
	950-800	-	-	-	-	-
IAGOS (WNA) (separate fit)	950-250	**4.98 [±3.83]**	0.01	-	-	-
	700-300	***3.97 [±1.80]***	<0.01	-	-	-
	400-300	***5.89 [±4.03]***	<0.01	-	-	-
	650	***2.34 [±1.56]***	<0.01	-	-	-
	950-800	0.24 [±2.14]	0.82	-	-	-
Fused (WNA)	700-250	**0.31 [±0.28]**	0.02	0.04 [±0.27]	0.76	-86
	700-300	***0.34 [±0.22]***	<0.01	0.11 [±0.22]	0.33	-68
	400-300	***0.64 [±0.39]***	<0.01	0.36 [±0.38]	0.06	-44
	650	***0.40 [±0.20]***	. <0.01	0.17 [±0.21]	0.10	-57
	950-800	-	-	-	-	-

Table S-2: Same as Table S1.

		Through 2019		Through 2020		
Site	Pressure [hPa]	Trend [±2-σ]	p-value	Trend [±2-σ]	p-value	Change [%]
Legionowo	950-250	***-1.67 [±1.02]***	<0.01	***-1.68 [±0.98]***	<0.01	-0
	700-300	***-1.52 [±1.02]***	<0.01	***-1.60 [±0.98]***	<0.01	-5
	400-300	-2.35 [±2.44]	0.06	**-2.80 [±2.31]**	0.02	-19
	650	**-0.92 [±0.74]**	0.01	**-0.97 [±0.72]**	0.01	-6
	950-800	-0.16 [±0.88]	0.71	-0.08 [±0.87]	0.86	53
Lindenberg	950-250	-1.94 [±2.50]	0.12	***-3.57 [±2.31]***	<0.01	-84
	700-300	-1.73 [±2.01]	0.09	***-2.85 [±1.83]***	<0.01	-65
	400-300	-1.59 [±4.88]	0.51	-3.57 [±4.34]	0.10	-124
	650	-0.82 [±1.78]	0.36	-1.41 [±1.56]	0.07	-72
	950-800	-0.22 [±1.78]	0.80	0.05 [±1.57]	0.95	122
De Bilt	950-250	***1.93 [±1.05]***	<0.01	***1.57 [±1.01]***	<0.01	-19
	700-300	***2.26 [±1.04]***	<0.01	***1.86 [±1.02]***	<0.01	-18
	400-300	**3.11 [±2.59]**	0.02	2.40 [±2.47]	0.05	-23
	650	***1.68 [±0.82]***	<0.01	***1.44 [±0.80]***	<0.01	-14
	950-800	**1.11 [±0.86]**	0.01	**1.05 [±0.81]**	0.01	-5
Uccle	950-250	***1.52 [±0.72]***	<0.01	***1.10 [±0.73]***	<0.01	-27
	700-300	***1.49 [±0.89]***	<0.01	**1.00 [±0.90]**	0.03	-33
	400-300	**1.76 [±1.49]**	0.02	1.12 [±1.45]	0.12	-36
	650	***1.19 [±0.69]***	<0.01	**0.80 [±0.71]**	0.02	-33
	950-800	***1.17 [±0.75]***	<0.01	***1.06 [±0.71]***	<0.01	-9
Hohenpeissenberg	950-250	-0.49 [±0.76]	0.20	**-0.78 [±0.78]**	0.05	-59
	700-300	-0.17 [±0.73]	0.63	-0.48 [±0.75]	0.20	-173
	400-300	-0.54 [±1.39]	0.44	-0.92 [±1.35]	0.17	-69
	650	-0.08 [±0.69]	0.81	-0.32 [±0.68]	0.36	-292
	950-800	**-0.97 [±0.76]**	0.01	**-1.02 [±0.73]**	0.01	-6
Payerne	950-250	***-1.26 [±0.85]***	<0.01	***-1.53 [±0.83]***	<0.01	-21
	700-300	***-1.56 [±0.85]***	<0.01	***-1.81 [±0.83]***	<0.01	-16
	400-300	**-1.86 [±1.56]**	0.02	***-2.29 [±1.50]***	<0.01	-23
	650	***-1.04 [±0.62]***	<0.01	***-1.17 [±0.60]***	<0.01	-13
	950-800	**-0.88 [±0.86]**	0.04	**-0.97 [±0.82]**	0.02	-10
OHP	950-250	0.37 [±1.16]	0.53	0.14 [±1.10]	0.80	-61
	700-300	**1.29 [±1.13]**	0.02	1.03 [±1.07]	0.06	-20
	400-300	**2.36 [±1.95]**	0.02	1.72 [±1.87]	0.07	-27
	650	0.58 [±1.10]	0.30	0.41 [±1.05]	0.44	-30
	950-800	***-2.26 [±1.17]***	<0.01	***-2.24 [±1.10]***	<0.01	1
Madrid	950-250	-0.50 [±1.34]	0.45	-0.96 [±1.28]	0.13	-91
	700-300	-0.39 [±1.34]	0.56	-0.88 [±1.29]	0.18	-124
	400-300	-0.63 [±2.72]	0.65	1.57 [±2.60]	0.23	-151
	650	-0.11 [±0.93]	0.82	-0.43 [±0.89]	0.33	-311
	950-800	-0.55 [±1.06]	0.30	-0.62 [±0.99]	0.21	-12
IAGOS (EU) (separate fit)	950-250	**0.98 [±0.81]**	0.02	**0.88 [±0.77]**	0.02	-10
	700-300	***1.16 [±0.77]***	<0.01	**1.02 [±0.75]**	0.01	-12
	400-300	***1.76 [±1.18]***	<0.01	**1.65 [±1.18]**	0.01	-7
	650	**0.76 [±0.68]**	0.03	0.54 [±0.65]	0.10	-28
	950-800	**0.76 [±0.70]**	0.03	**0.83 [±0.66]**	0.01	9
Fused (EU) (final)	950-250	***0.60 [±0.20]***	<0.01	***0.35 [±0.20]***	<0.01	-42
	700-300	***0.65 [±0.19]***	<0.01	***0.36 [±0.20]***	<0.01	-44
	400-300	***1.08 [±0.27]***	<0.01	***0.66 [±0.29]***	<0.01	-39
	650	***0.47 [±0.18]***	<0.01	**0.25 [±0.19]**	0.01	-47
	950-800	-0.03 [±0.21]	0.81	-0.05 [±0.20]	0.61	-99

Table S-3: Same as Table S1.

		Through 2019		Through 2020		
Site	Pressure [hPa]	Trend [±2-σ]	p-value	Trend [±2-σ]	p-value	Change [%]
Broadmeadows	950-250	***-2.32 [±1.28]***	<0.01	***-1.93 [±1.20]***	<0.01	17
	700-300	-1.07 [±1.26]	0.09	-0.71 [±1.18]	0.23	34
	400-300	-1.98 [±2.53]	0.12	-1.65 [±2.32]	0.16	17
	650	0.37 [±1.08]	0.49	0.79 [±1.09]	0.15	112
	950-800	0.02 [±1.04]	0.97	0.01 [±0.98]	0.98	-34
Lauder	950-250	0.14 [±0.70]	0.70	0.08 [±0.66]	0.80	-38
	700-300	0.34 [±0.77]	0.38	0.27 [±0.73]	0.46	-21
	400-300	-0.05 [±2.22]	0.97	-0.22 [±2.07]	0.83	-370
	650	**0.61 [±0.55]**	0.03	**0.60 [±0.52]**	0.02	-1
	950-800	**0.39 [±0.32]**	0.01	**0.39 [±0.30]**	0.01	-1
Macquarie Island	950-250	***-8.68 [±2.33]***	<0.01	***-7.90 [±2.22]***	<0.01	9
	700-300	***-4.23 [±1.96]***	<0.01	***-3.74 [±1.85]***	<0.01	11
	400-300	**-3.88 [±2.95]**	0.01	**-3.31 [±2.78]**	0.02	15
	650	***-1.31 [±0.81]***	<0.01	**-0.98 [±0.77]**	0.01	26
	950-800	**-0.97 [±0.84]**	0.02	**-0.80 [±0.78]**	0.04	17
Tateno	950-250	-0.29 [±6.32]	0.93	-2.29 [±5.33]	0.39	-696
	700-300	-0.58 [±6.32]	0.85	-2.15 [±5.36]	0.42	-269
	400-300	1.17 [±7.54]	0.76	-1.72 [±6.43]	0.59	-247
	650	-3.36 [±8.90]	0.45	-4.70 [±7.31]	0.20	-40
	950-800	0.13 [±6.80]	0.97	-1.66 [±5.67]	0.56	-1379
Izaña	950-250	***2.64 [±0.89]***	<0.01	***2.29 [±0.87]***	<0.01	-13
	700-300	***3.01 [±1.00]***	<0.01	***2.65 [±0.97]***	<0.01	-12
	400-300	***3.16 [±1.66]***	<0.01	***2.57 [±1.62]***	<0.01	-19
	650	***3.11 [±1.66]***	<0.01	***2.57 [±1.57]***	<0.01	-17
	950-800	***1.94 [±0.77]***	<0.01	***1.83 [±0.73]***	<0.01	-6
Hong Kong	950-250	1.20 [±2.54]	0.34	1.14 [±2.35]	0.33	-5
	700-300	0.54 [±2.66]	0.69	0.55 [±2.45]	0.66	1
	400-300	0.10 [±3.14]	0.95	0.14 [±2.88]	0.92	41
	650	1.20 [±3.27]	0.46	1.15 [±3.04]	0.45	-4
	950-800	2.90 [±3.01]	0.05	2.65 [±2.79]	0.06	-9
Hilo	950-250	0.56 [±1.22]	0.36	0.42 [±1.15]	0.47	-26
	700-300	0.75 [±1.45]	0.30	0.67 [±1.35]	0.32	-11
	400-300	0.80 [±2.08]	0.44	0.80 [±1.94]	0.41	-0.1
	650	-0.23 [±1.33]	0.73	-0.21 [±1.25]	0.74	8
	950-800	0.21 [±0.97]	0.67	-0.09 [±0.94]	0.85	-144

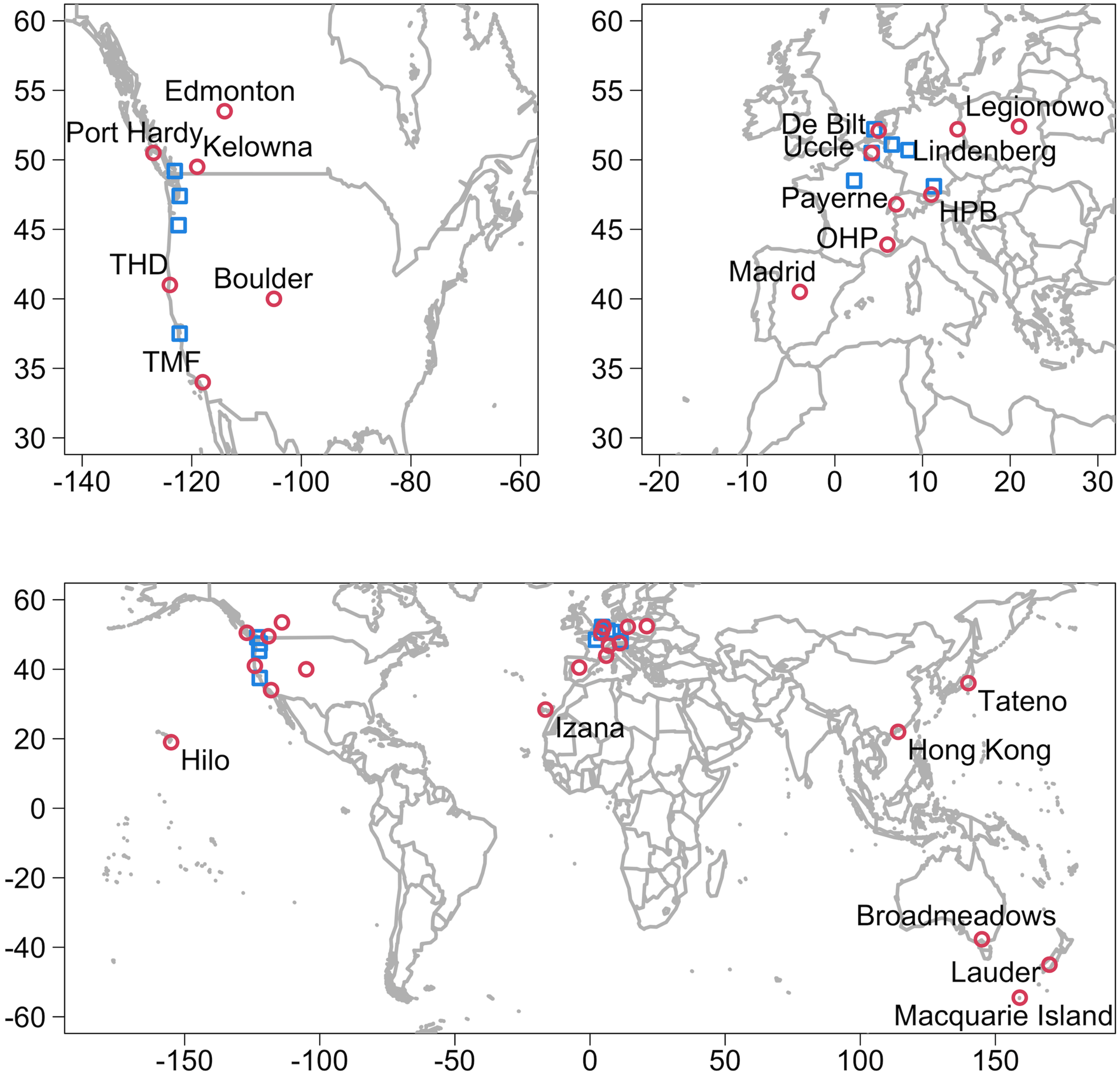

Figure S-1: A map view of ozonesonde or lidar stations (red circles), and the major airports visited by IAGOS aircraft in Europe and western North America (blue boxes).

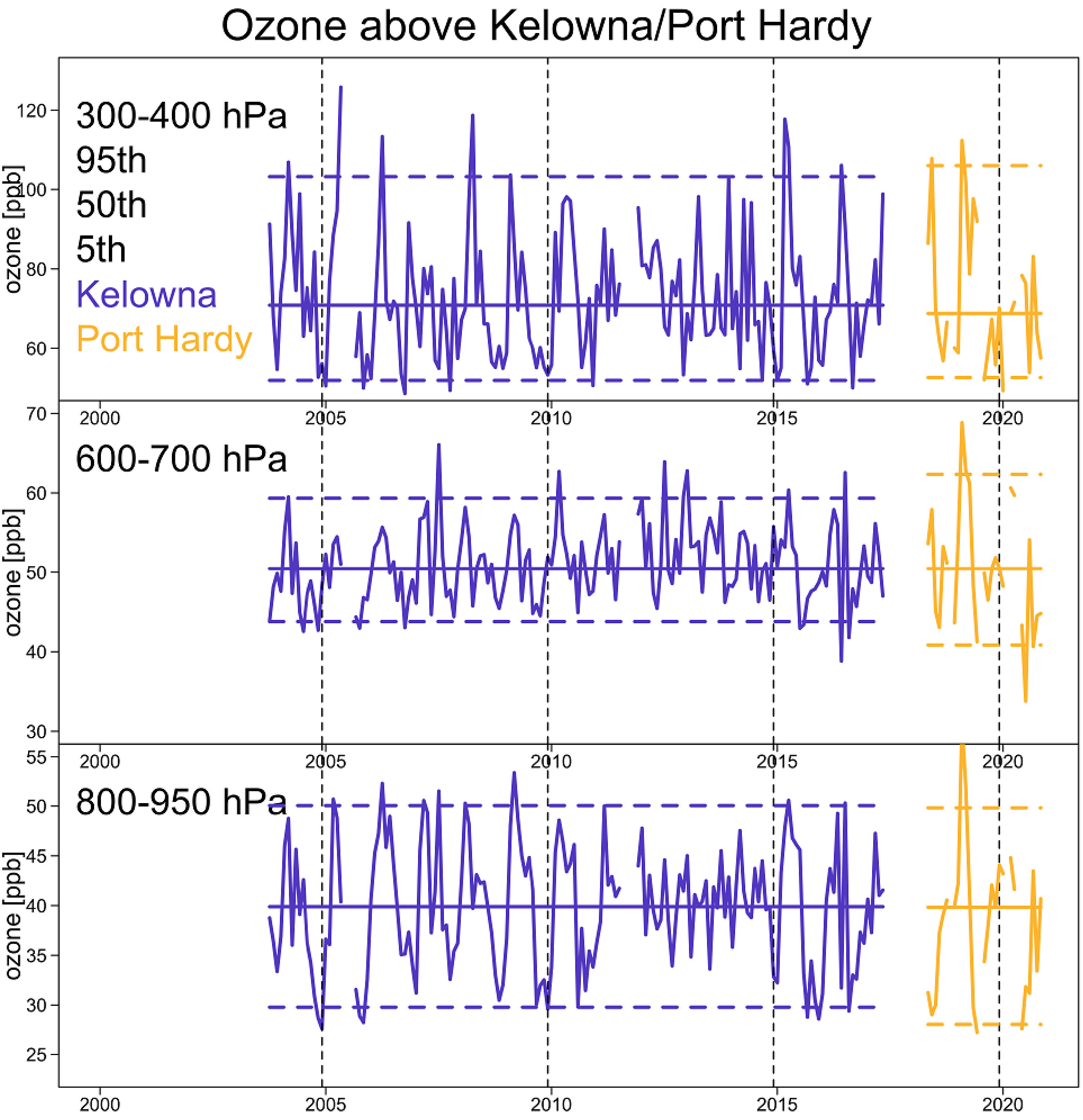

Figure S-2: Comparisons of ozone time series at Kelowna (2003-2017) and Port Hardy (2018-2020) based on the 95th, 50th and 5th percentiles at different pressure layers.

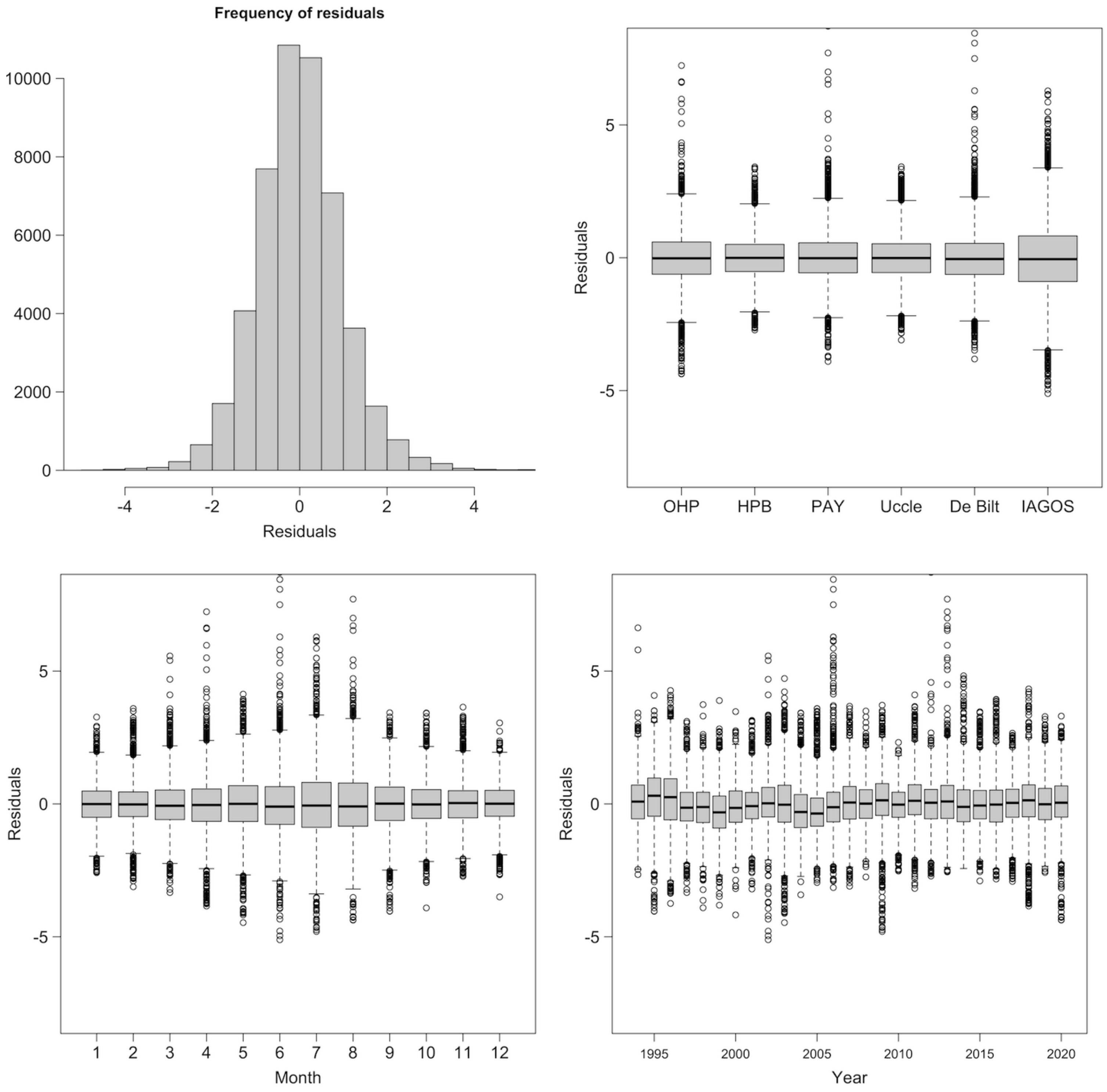

Figure S-3: Residuals diagnostics of the tropospheric ozone data fusion above Western Europe.

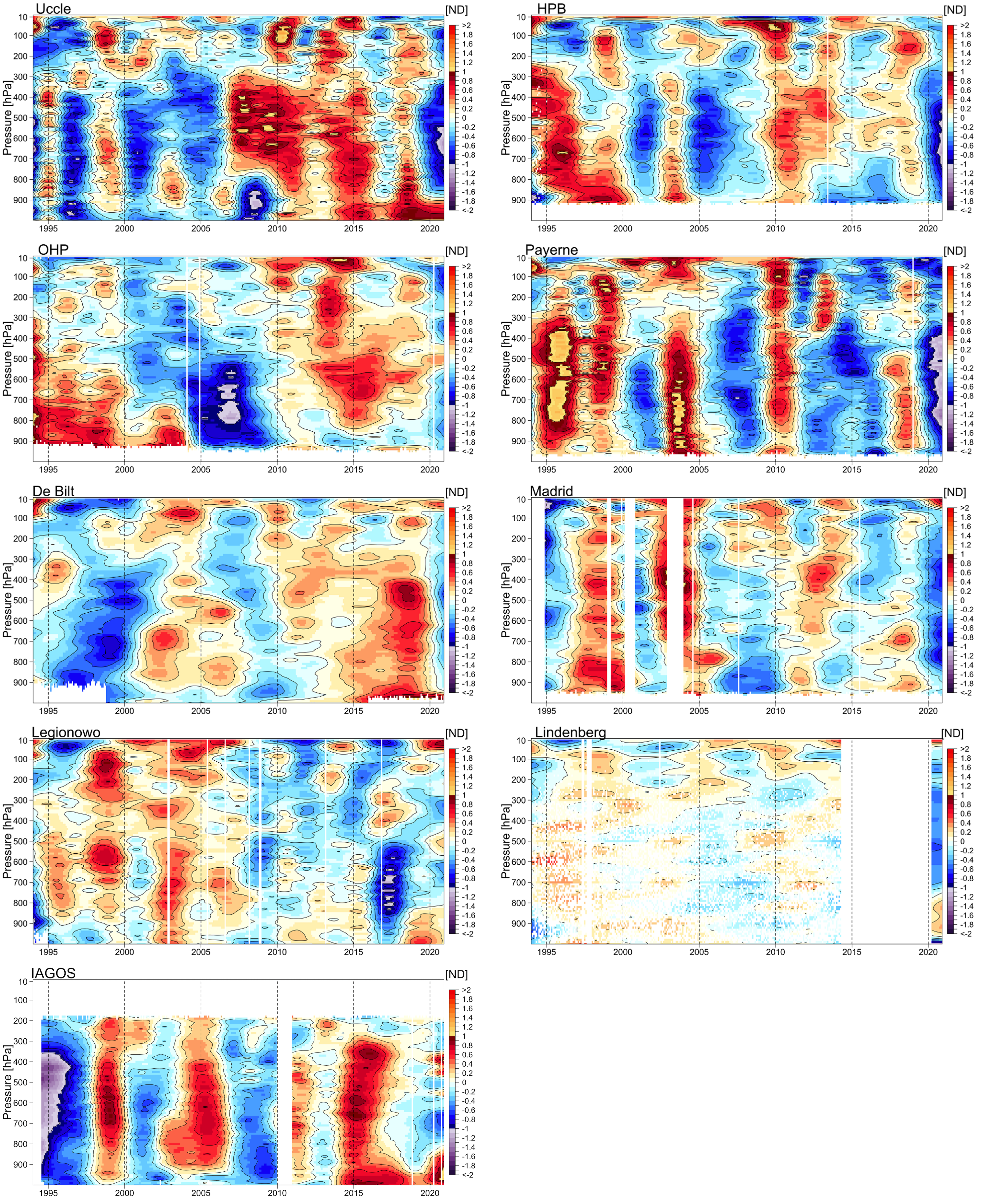

Figure S-4: Ozone mean distributions above Western Europe based on the normalized deviations.

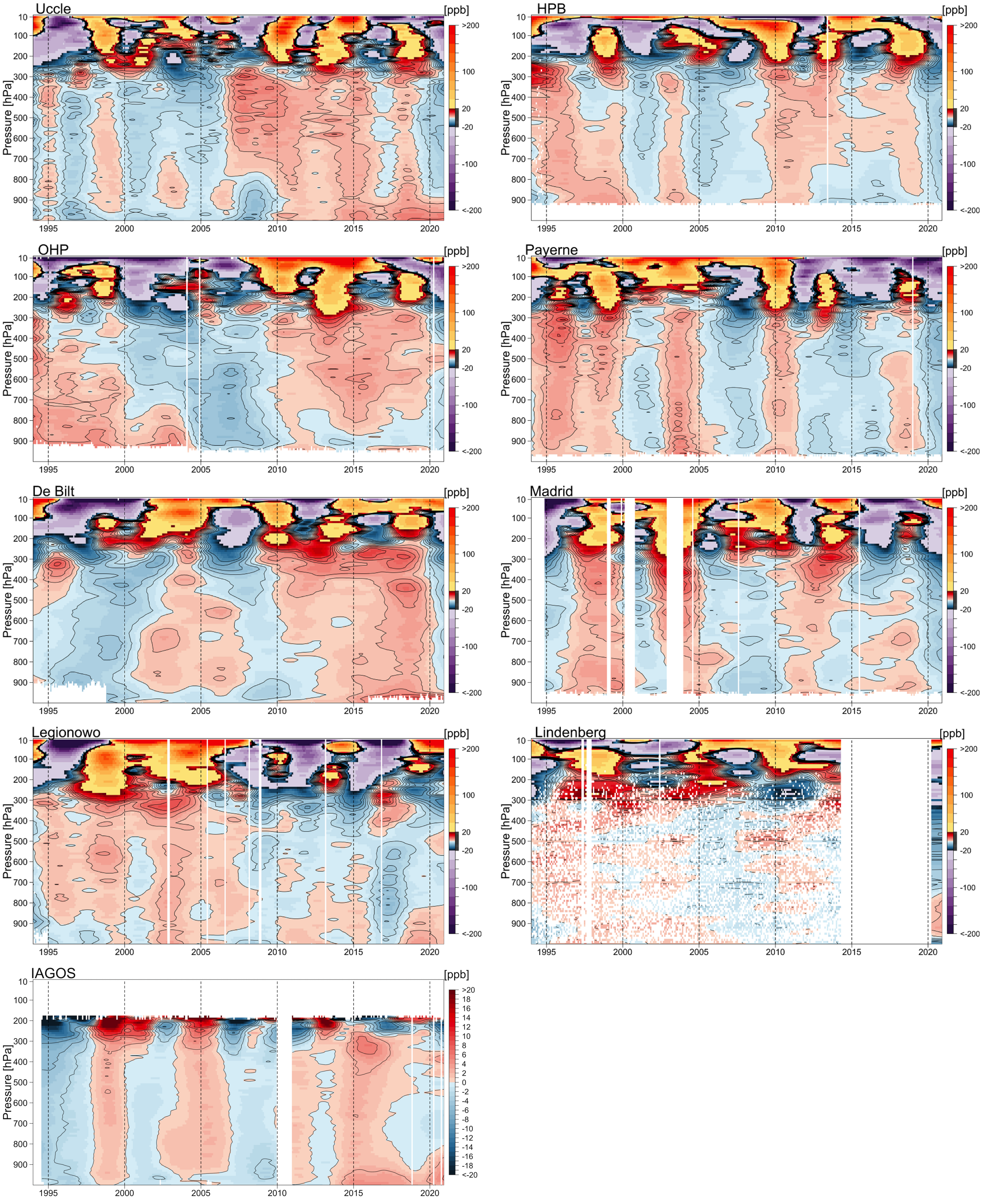

Figure S-5: Same as Figure S4, but ozone mean distributions are transformed back to the units of ppbv.

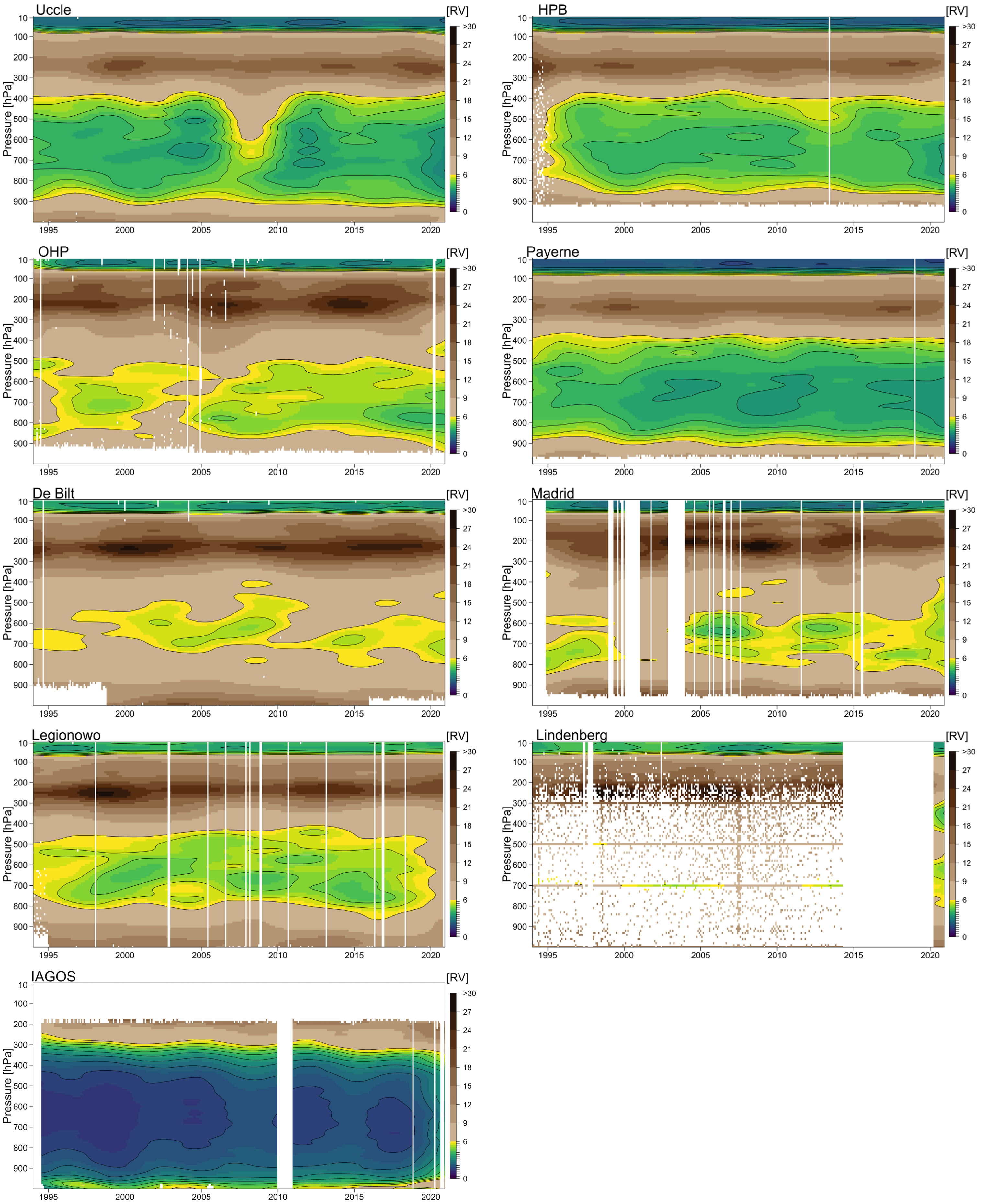

Figure S-6: Relative variability (RV) from each ozonesonde record and IAGOS data above Western Europe. The estimations are based on the standard error associated with each monthly aggregated mean divided by the overall mean at the corresponding pressure surface.

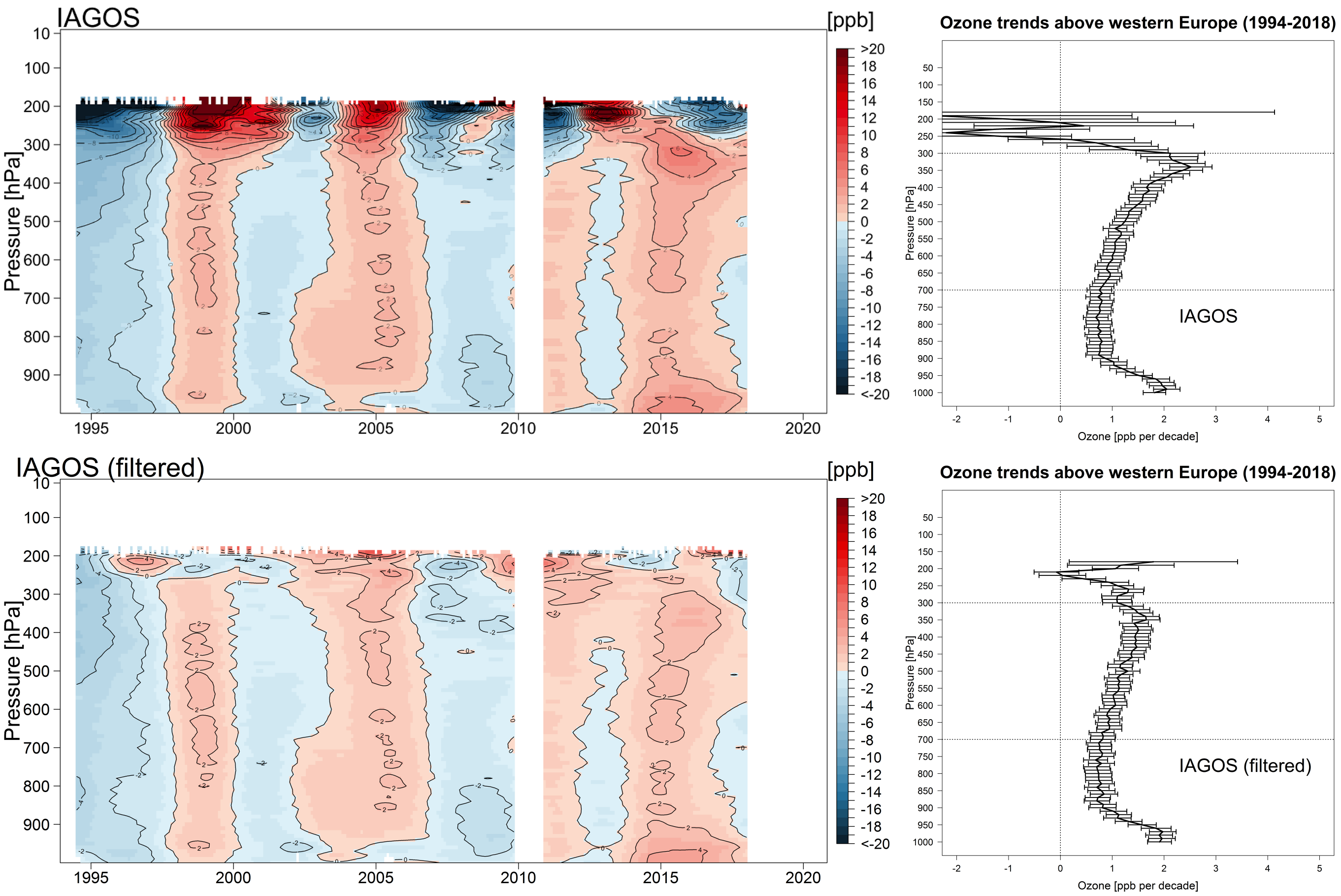

Figure S-7: A demonstration of the influence of stratosphere-troposphere exchange on tropospheric ozone trends above Europe over 1994-2018. The first row shows the vertical distribution and trends, when the stratospheric air masses are included. The second row is the same as the first row, but the stratospheric air masses are removed.

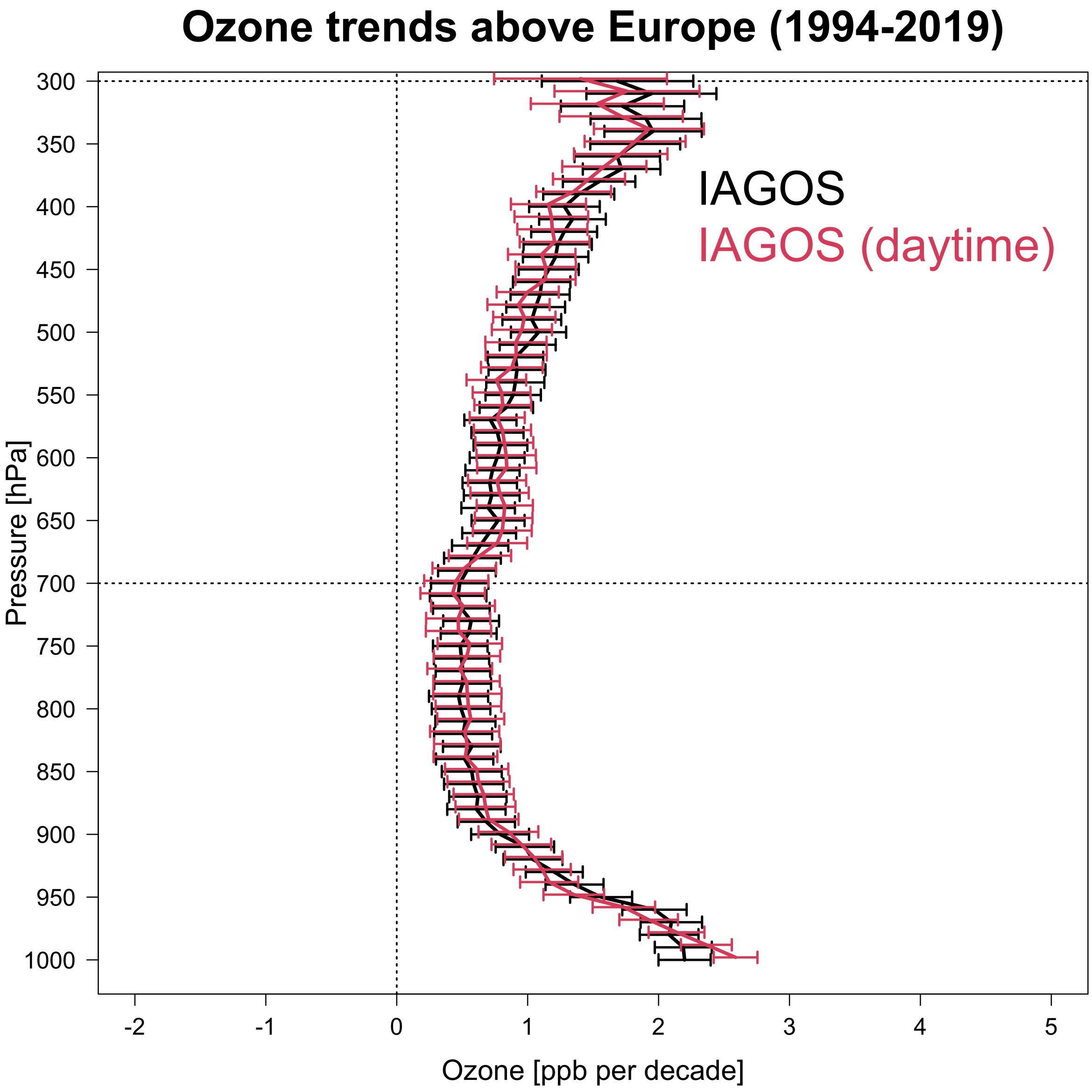

,

Figure S-8: A demonstration of the influence of diurnal cycle on tropospheric ozone trends above Europe, by comparing the trends in the European IGAOS data set with and without the nighttime observations.

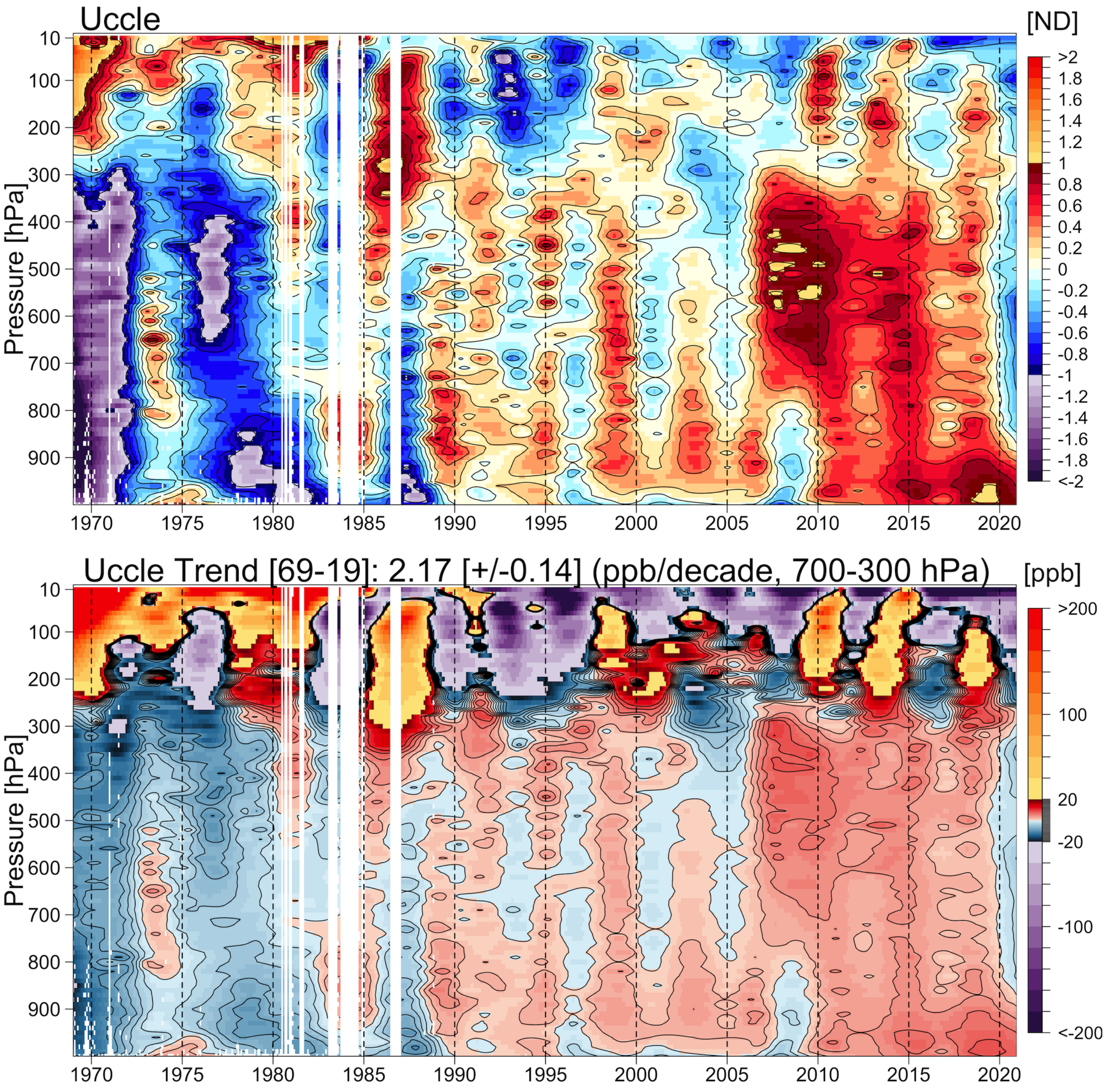

Figure S-9: Ozone mean distributions above Uccle over 1969-2020 based on normalized deviation [ND] and the units of ppbv.

The free tropospheric trend [ppbv/decade, 700-300 hPa] was 2.17 [±0.14, $p<0.01$] for integrated fit and 2.18 [±0.43, $p<0.01$] for separated fit over 1969-2019; 2.01 [±0.14, $p<0.01$] for integrated fit and 2.02 [±0.43, $p<0.01$] for separated fit over 1969-2020 (see Chang et al. (2020)).

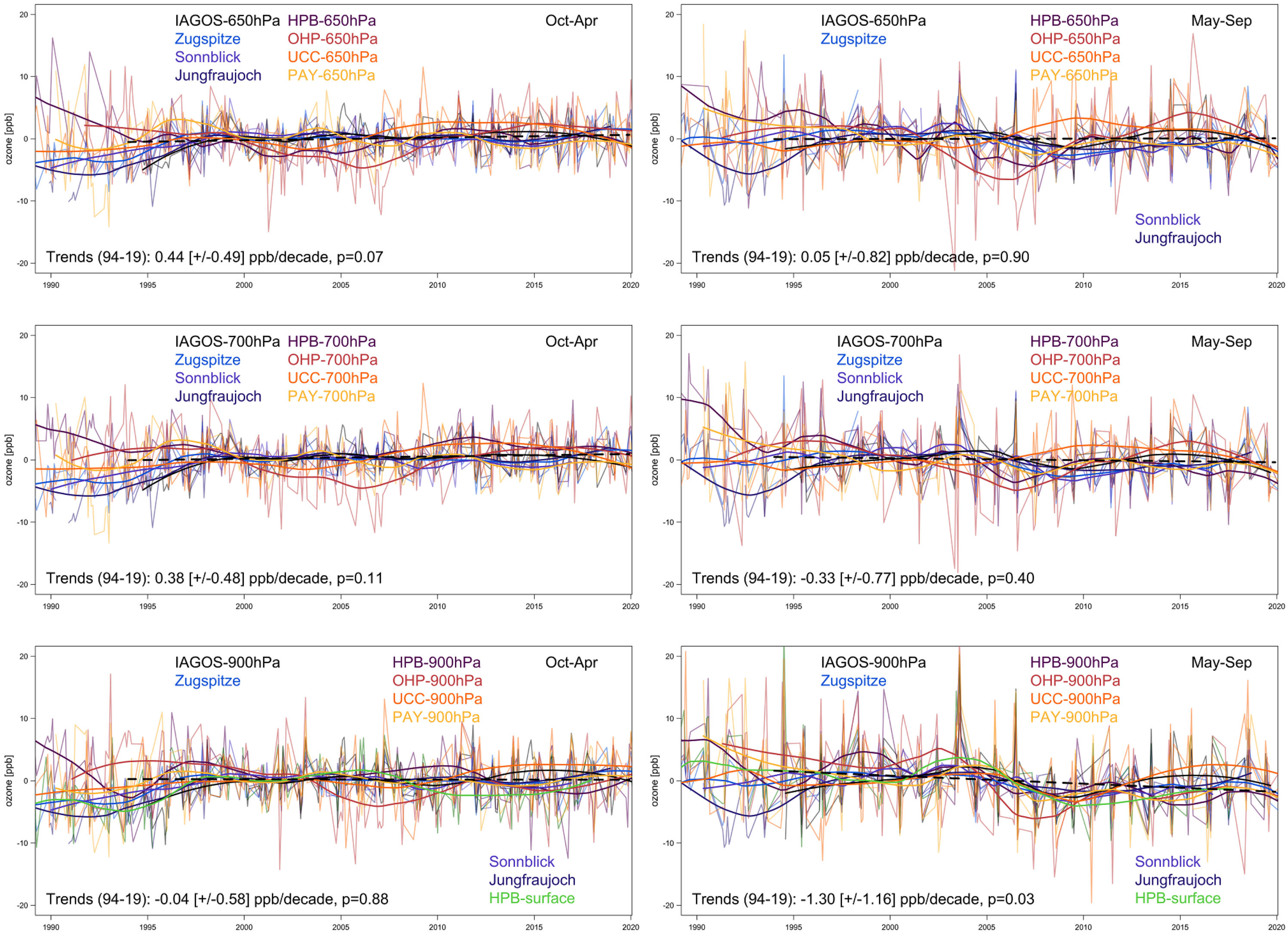

Figure S-10: Comparison of monthly anomalies from ozonesondes and surface measurements limited within the cold (Oct-Apr) or warm (May-Sep) season at different pressure surfaces in Western Europe. The overall trends are based on the simple average of ozonesonde and IAGOS data (not including the surface measurements).

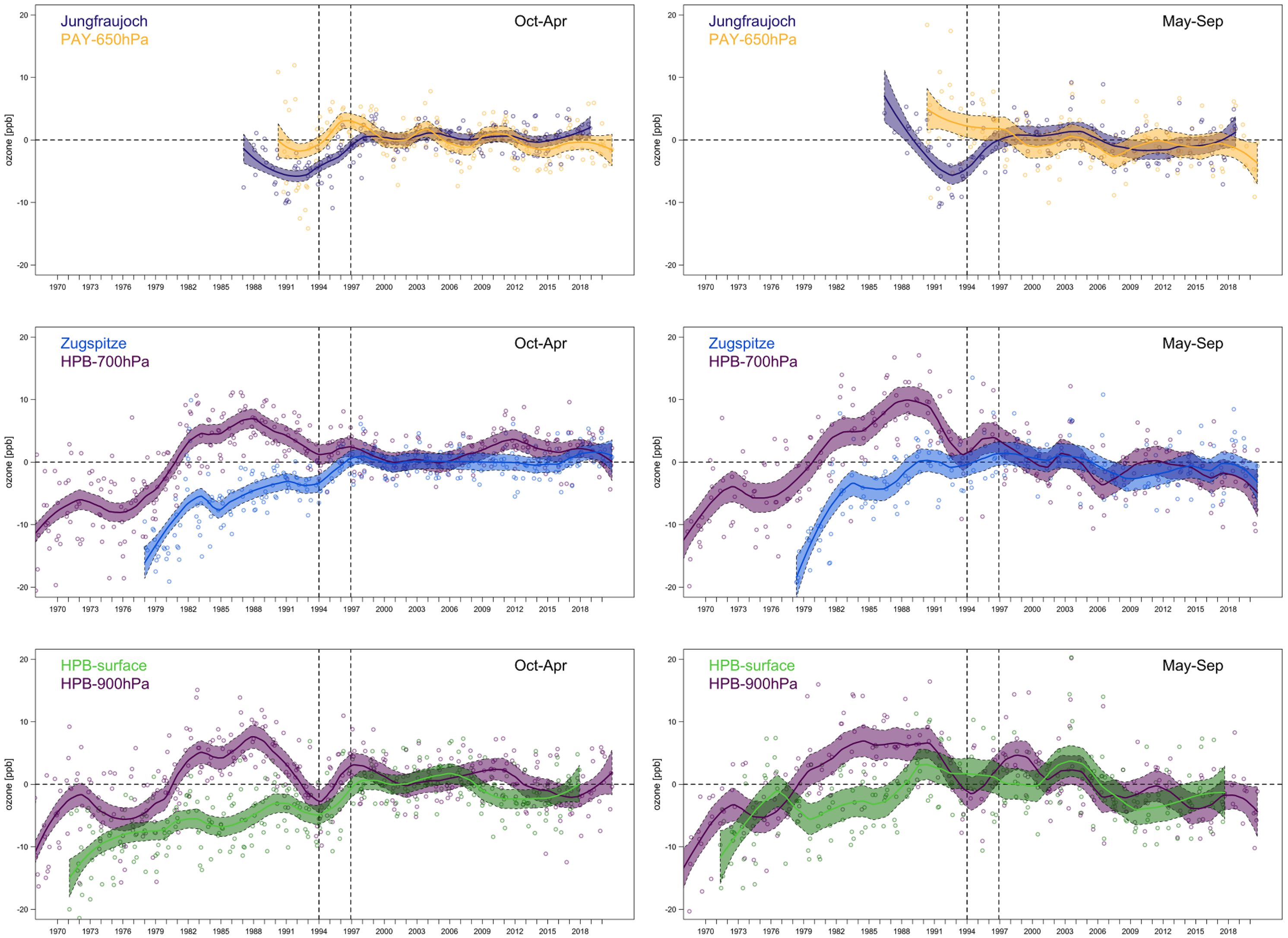

Figure S-11: Comparisons of monthly anomalies from ozonesonde and surface measurements limited within the cold (Oct-Apr) or warm (May-Sep) season at different pressure surfaces (1970-2020). The smooth curves represent the Loess fits and their 2-sigma intervals.

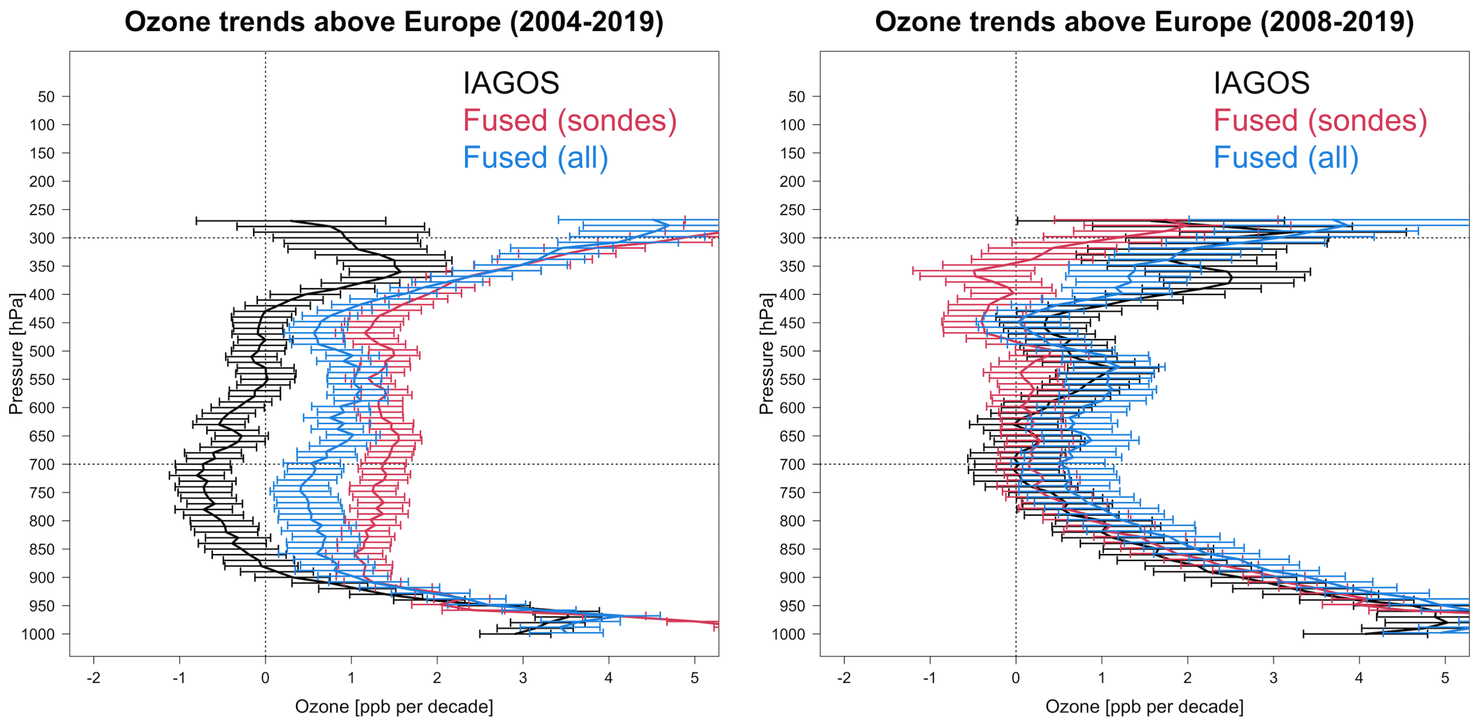

Figure S-12: Ozone mean trends above Western Europe [in units of ppbv/decade] derived from the fused product over 2004-2019 (left panel) and 2008-2019 (right panel).

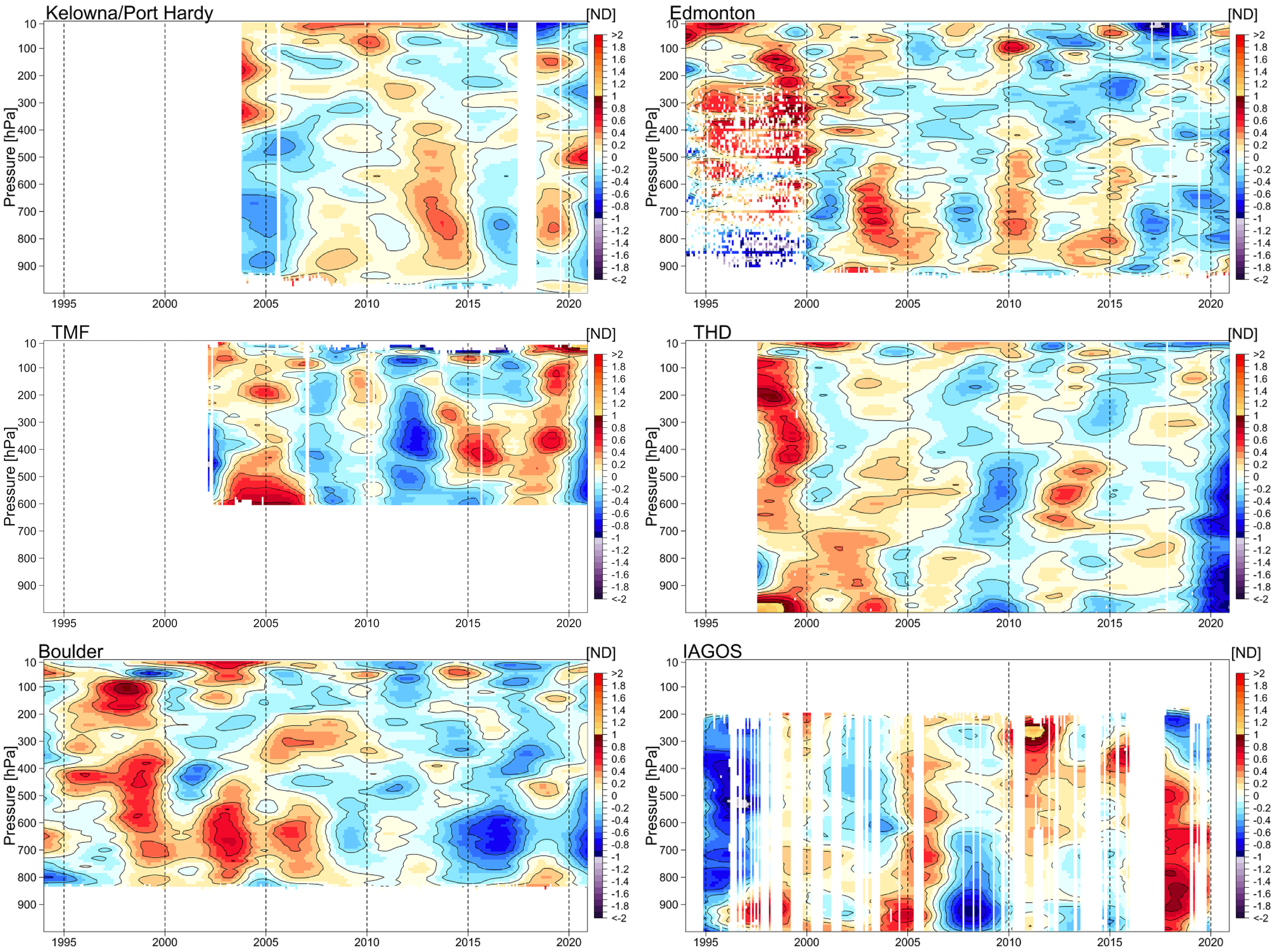

Figure S-13: Ozone mean distributions above western North America based on the normalized deviations.

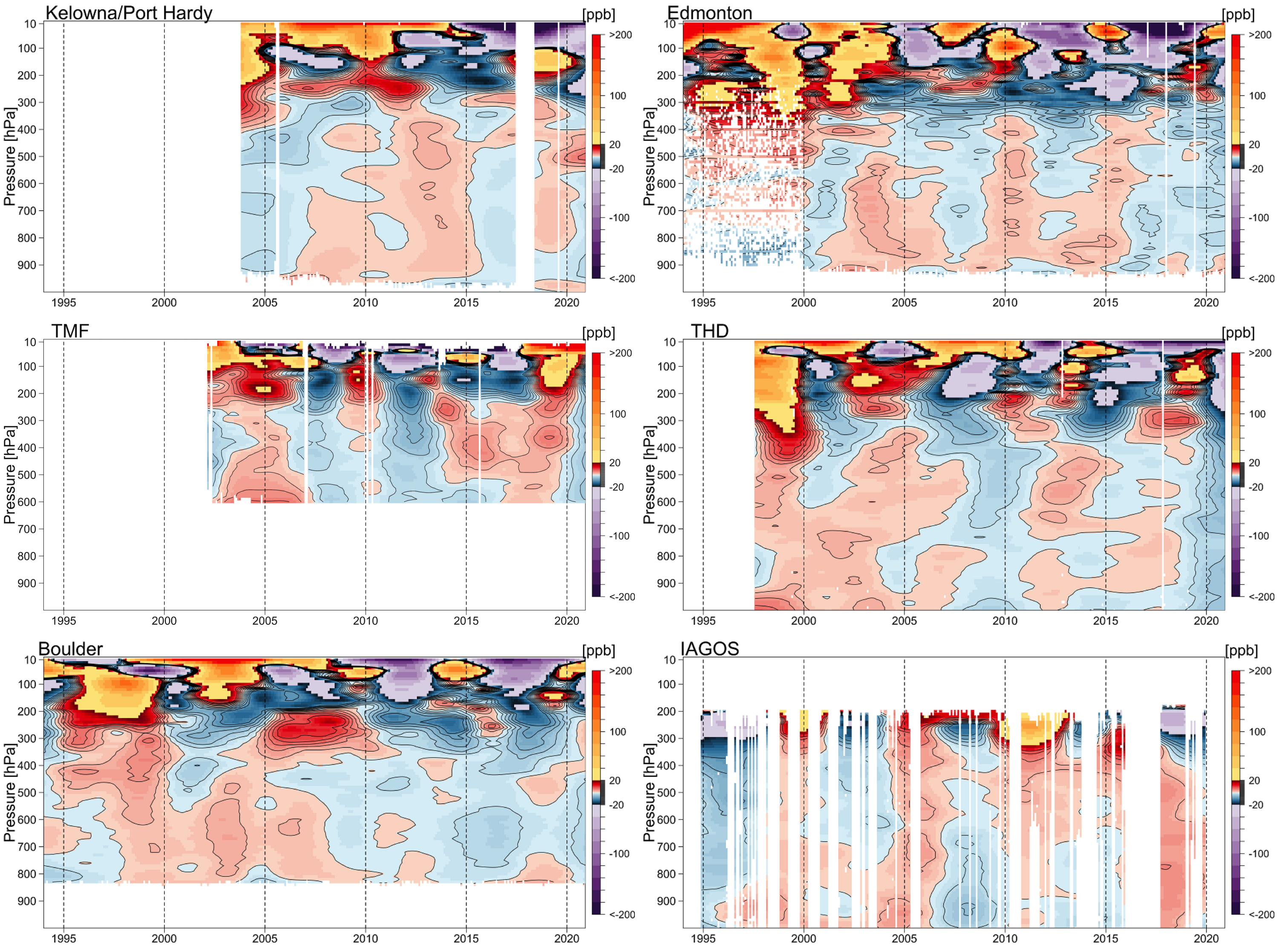

Figure S-14: Same as Figure S13, but ozone mean distributions are transformed back to the units of ppbv.

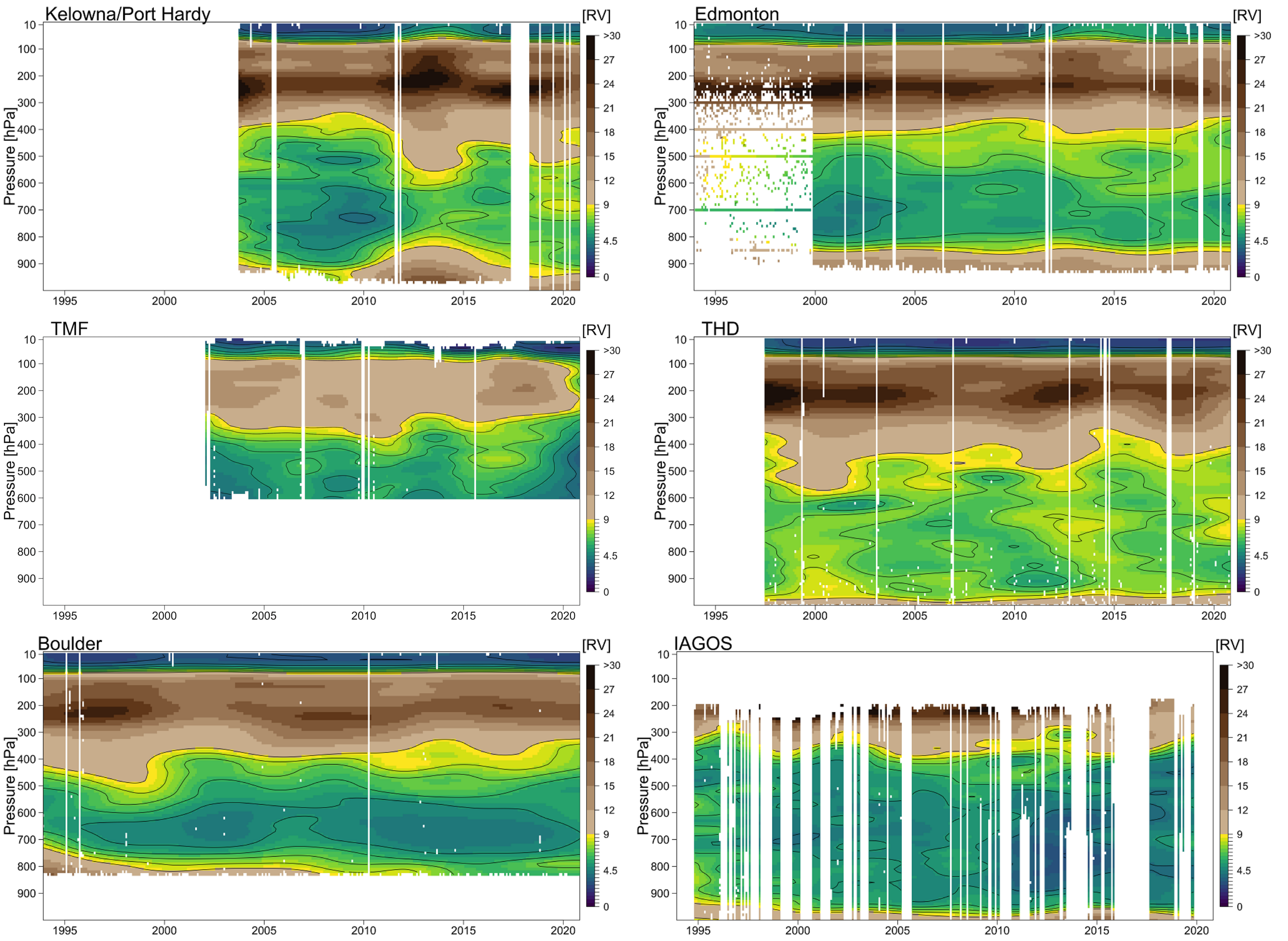

Figure S-15: Relative variability (RV) from each ozonesonde record and IAGOS data above western North America. The estimations are based on the standard error associated with each monthly aggregated mean divided by the overall mean at the corresponding pressure surface.

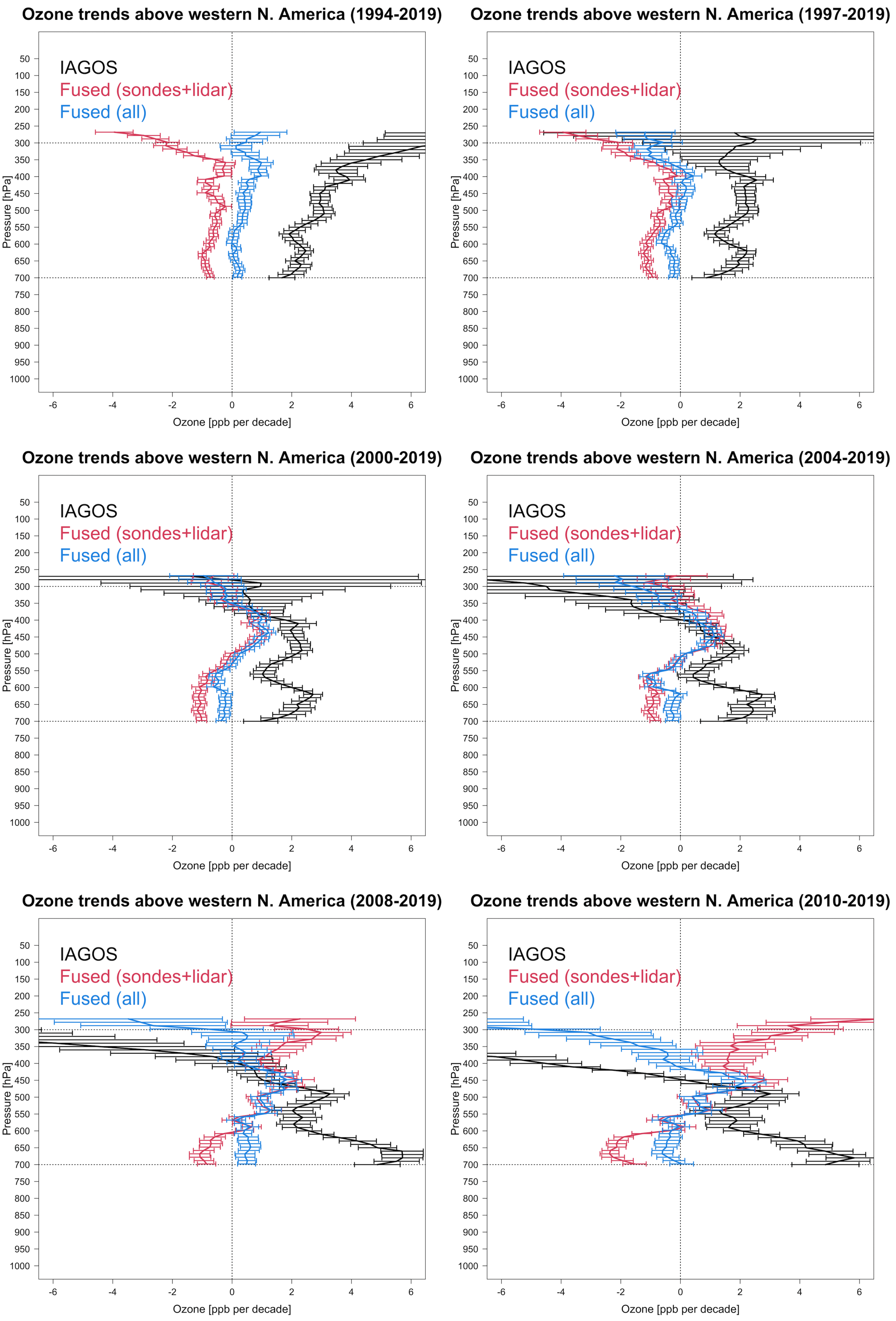

Figure S-16: Ozone mean trends above western N. America [in units of ppbv/decade] derived from the fused product over different periods.

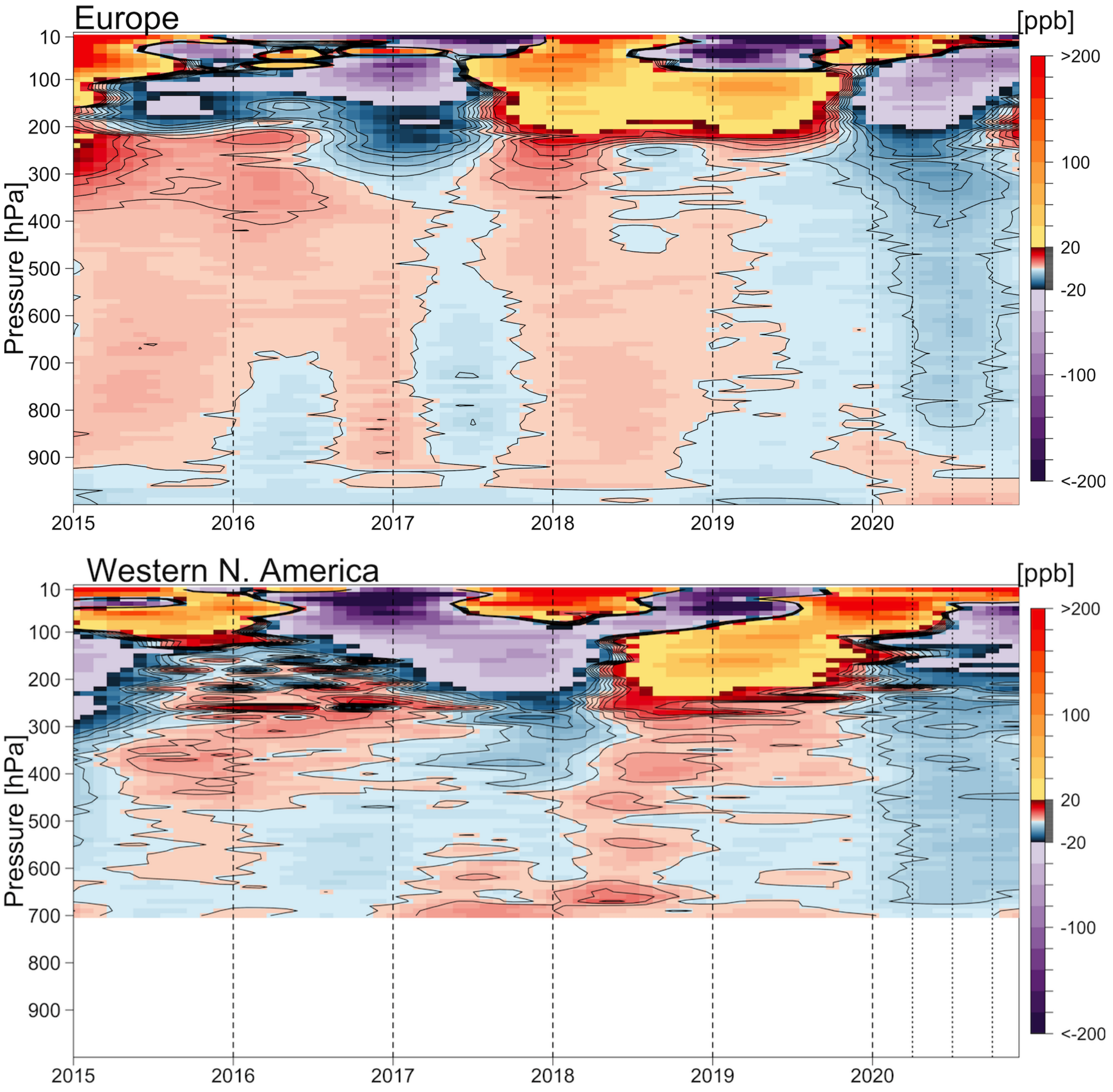

Figure S-17: Detailed inspection of 2020 ozone anomalies [in units of ppbv] above western Europe and western North America, limited to the period of 2015-2020.

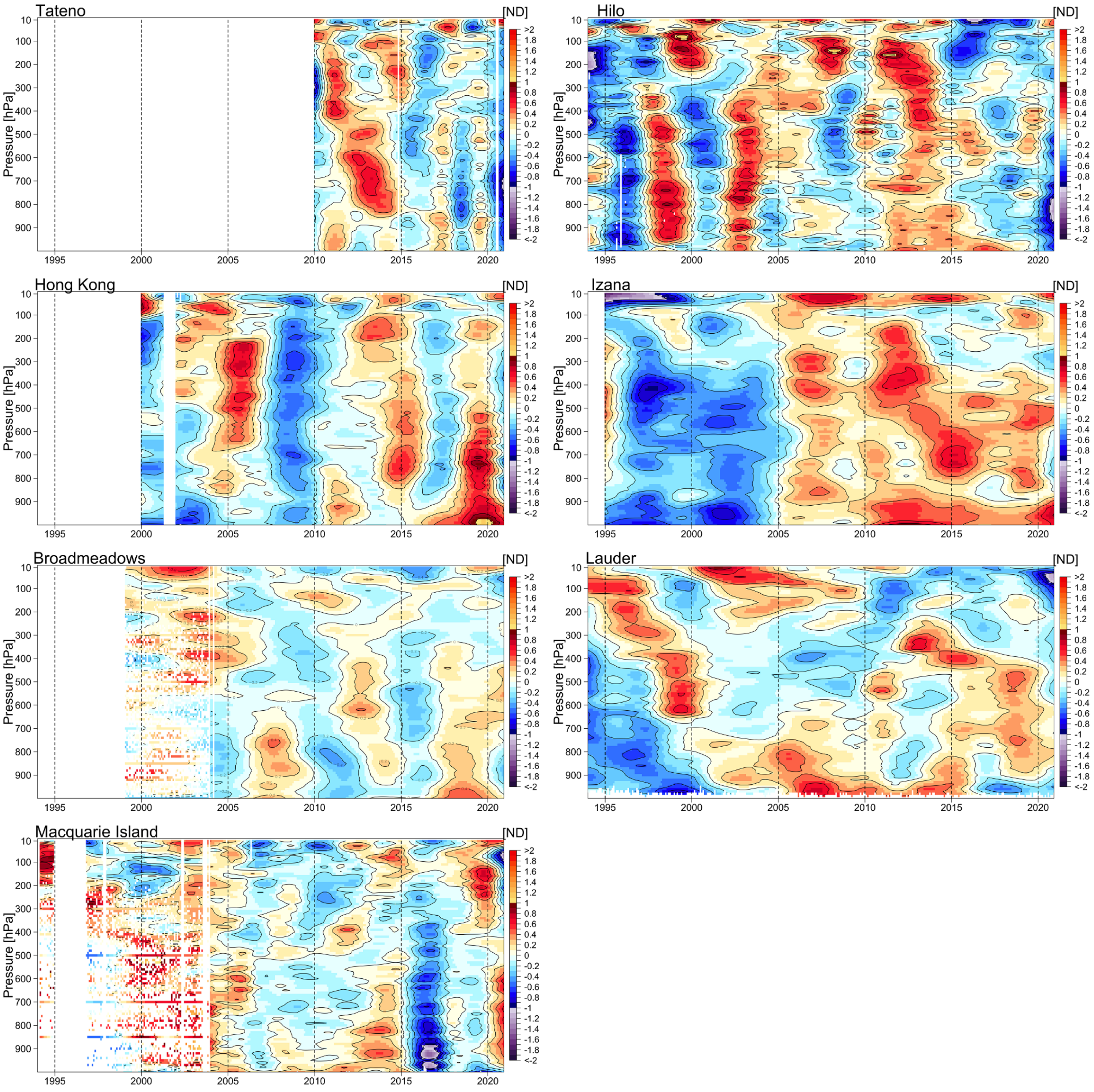

Figure S-18: Ozone mean distributions based on the normalized deviations.

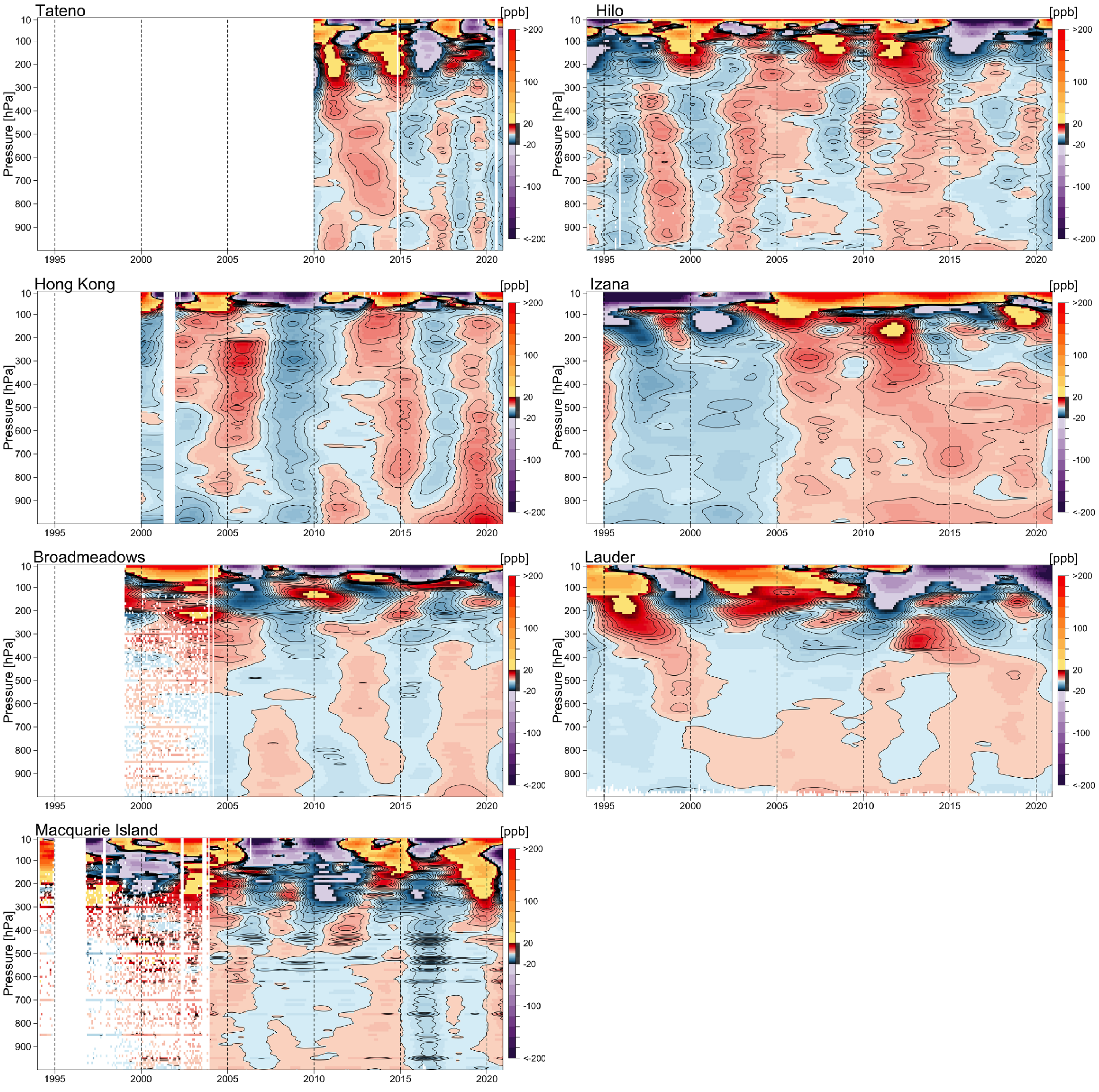

Figure S-19: Same as Figure S18, but ozone mean distributions are transformed back to the units of ppbv.

References

Chang, K.-L., Cooper, O. R., Gaudel, A., Petropavlovskikh, I., and Thouret, V. (2020). Statistical regularization for trend detection: An integrated approach for detecting long-term trends from sparse tropospheric ozone profiles. *Atmos. Chem. Phys.*

Cooper, O. R., Schultz, M. G., Schröder, S., Chang, K.-L., Gaudel, A., Benitez, G. C., Cuevas, E., Fröhlich, M., Galbally, I. E., Molloy, S., Kubistin, D., Lu, X., McClure-Begley, A., Nédélec, P., O'Brien, J., Oltmans, S. J., Petropavlovskikh, I., Ries, L., Senik, I., Sjöberg, K., Solberg, S., Spain, G. T., Steinbacher, M., Tarasick, D. W., Thouret, V., and Xu, X. (2020). Multi-decadal surface ozone trends at globally distributed remote locations. *Elem. Sci. Anth.*, 8(23).

Gaudel, A., Cooper, O. R., Ancellet, G., Barret, B., Boynard, A., Burrows, J. P., Clerbaux, C., Coheur, P. F., Cuesta, J., Cuevas, E., Doniki, S., Dufour, G., Ebojie, F., Foret, G., Garcia, O., Muños, M. J. G., Hannigan, J. W., Hase, F., Huang, G., Hassler, B., Hurtmans, D., Jaffe, D., Jones, N., Kalabokas, P., Kerridge, B., Kulawik, S. S., Latter, B., Leblanc, T., Flochmoën, E. L., Lin, W., Liu, J., Liu, X., Mahieu, E., McClure-Begley, A., Neu, J. L., Osman, M., Palm, M., Petetin, H., Petropavlovskikh, I., Querel, R., Rahpoe, N., Rozanov, A., Schultz, M. G., Schwab, J., Siddans, R., Smale, D., Steinbacher, M., Tanimoto, H., Tarasick, D. W., Thouret, V., Thompson, A. M., Trickl, T., Weatherhead, E. C., Wespes, C., Worden, H. M., Vigouroux, C., Xu, X., Zeng, G., and Ziemke, J. R. (2018). Tropospheric Ozone Assessment Report: Present-day distribution and trends of tropospheric ozone relevant to climate and global atmospheric chemistry model evaluation. *Elem. Sci. Anth.*, 6(39).

Oltmans, S. J., Lefohn, A. S., Shadwick, D., Harris, J. M., Scheel, H. E., Galbally, I., Tarasick, D. W., Johnson, B. J., Brunke, E.-G., Claude, H., Zeng, G., Nichol, S., Schmidlin, F., Davies, J., Cuevas, E., Redondas, A., Naoe, H., Nakano, T., and Kawasato, T. (2013). Recent tropospheric ozone changes–a pattern dominated by slow or no growth. *Atmos. Environ.*, 67:331–351.

Wood, S. N. (2006). *Generalized additive models: an introduction with R.* CRC press, New York, USA.
